# Effects of Probiotics on Anxiety, Stress, Mood and Fitness of Badminton Players

**DOI:** 10.3390/nu13061783

**Published:** 2021-05-24

**Authors:** Razali Mohamed Salleh, Garry Kuan, Mohd Noorazlan Ab Aziz, Mohamad Rahizam Abd Rahim, Tandiyo Rahayu, Sulaiman Sulaiman, Donny Wira Yudha Kusuma, A.M.G.C.P. Adikari, Muhammad Syafiq Mohd Razam, Ammu K. Radhakrishnan, Mahenderan Appukutty

**Affiliations:** 1 Sports Science Programme, Faculty of Sports Science and Recreation, Universiti Teknologi MARA, Shah Alam 40450, Malaysia; razalims@uitm.edu.my (R.M.S.); mohdnoorazlan@uitm.edu.my (M.N.A.A.); mrahizam@uitm.edu.my (M.R.A.R.); chandima@sjp.ac.lk (A.M.G.C.P.A.); muhammadsfiq10@gmail.com (M.S.M.R.); 2 Exercise and Sports Science Programme, School of Health Sciences, Universiti Sains Malaysia, Kubang Kerian Kelantan 16150, Malaysia; 3 Department of Life Sciences, Brunel University, London UB8 3PH, UK; 4 Faculty of Sports Science, Universitas Negeri Semarang, Semarang 50229, Indonesia; tandiyorahayu@mail.unnes.ac.id (T.R.); sulaiman@mail.unnes.ac.id (S.S.); donnywirayudhakusuma@mail.unnes.ac.id (D.W.Y.K.); 5 Department of Sports Science, Faculty of Applied Sciences, University of Sri Jayewardenepura, Nugegoda 10250, Sri Lanka; 6 Jeffrey Cheah School of Medicine and Health Sciences, Monash University Malaysia, Sunway 47500, Malaysia; ammu.radhakrishnan@monash.edu

**Keywords:** probiotics, anxiety, stress, mood, aerobic capacity, badminton players

## Abstract

Background: Reports of probiotic consumption on athletes’ performance are debatable due to their equivocal results. There is a need for more evidence on the effects of probiotic intake on psychological state and fitness level. Thus, this study determined the effects of daily probiotic consumption on competitive anxiety, perceived stress and mood among university badminton players, besides their fitness like aerobic capacity, strength, speed, leg power and agility. Methods: Thirty university badminton players aged from 19 to 22 years old were randomly divided equally into two groups, where the probiotic group (PG; *n* = 15) received a drink that contained *Lactobacillus casei* Shirota (3 × 10^10^ CFU) and placebo group (CG; *n* = 15) a placebo drink for six weeks. Anxiety, stress and mood levels were determined using the CSAI-2R, PSS and BRUMS questionnaires, respectively. Fitness levels were measured using by subjecting the players to 20-m shuttle runs (aerobic capacity), handgrips (muscular strength), vertical jumps (leg power), 40-m dash (speed) and T-test (agility). The Student’s *t*-test (*p* < 0.05) was used to determine the differences between PG and CG players. Results: After six weeks, the anxiety and stress levels of PG players significantly decreased by 16% (*p* < 0.001) and 20% (*p* < 0.001), respectively, but there were no significant changes detected in CG players. Supplementation of probiotics also improved aerobic capacity in PG players by 5.9% (*p* < 0.001) but did not influence the speed, strength, leg power and agility. Conclusions: Probiotics supplementation showed improved aerobic capacity and relieve anxiety and stress. However, further studies need to be carried out to determine the mechanisms through which probiotic intake produces these effects.

## 1. Introduction

Exercise has shown evidence to improve fitness, wellbeing, and health [1]. However, strenuous exercise may be detrimental physiologically, but it may also affect the person psychologically [2]. Although high-performance athletes may not clinically diagnose with immune deficiency, there is evidence that prolonged and intense exercise may suppress the immune system, and impair sports performance [3]. Extreme exercise is also associated with psychological distress, as clinical evidence has shown that strenuous training may induce anxiety and stress [4].

On the other hand, exercise performed at a particular duration and intensity has a potential impact on gastrointestinal health [5]. Gut microbiota may significantly influence human health by forming a healthy microbiome in the digestive system that benefits the host. The effects of gut microbiota on health and sports performance depend on their composition [6]. Studies have shown that diet (i.e., the type, amount, and ratio of macronutrients) significantly impacts microbiota composition and metabolism [7]. Appropriate dietary choices are necessary to minimise the risk of gastrointestinal (GI) distress in competitive athletes by ensuring rapid gastric emptying, optimal absorption of water and nutrients, and sufficient splanchnic vasculature perfusion before competitions [8]. Supplementation is also part of the diet that may have a direct influence on the microbiota. Probiotics are some of the most common supplements to improve health by supplying “friendly” bacteria in the GI tract [9]. The common bacteria in probiotics comprise those from the *Lactobacillus* and *Bifidobacterium* genera that can provide health benefits for the host if taken in an adequate amount regularly [10].

Prolonged and stressful athletic training may increase the risk of injury, infection and depression, such as upper respiratory tract infections, gastrointestinal discomfort and psychological disorders [10,11]. The terms “anxiety”, “anticipation,” and “stress” are interrelated, where stress is described as the process by which a person perceives danger and reacts with a set of psychological and physiological changes, which may include increased anticipation and anxiety [12]. Anxiety is a negative emotion characterised by nervousness, over-concern and apprehension, which correlates with a sympathetic fight-or-flight response. Both conditions may excite the psychophysiology aspects of the human body. Mood swings and stress are common problems in athletes due to competitive stress [13]. The mood is an ephemeral collection of feelings in nature that varies in intensity and period and normally includes more than one emotion [14].

The benefits of taking probiotics to improve sports performance have been showcased by many investigators [6]. There are many ways to determine the effects of probiotics on physical performance. The level of fatigue can be measured by recording the running time to exhaustion [15]. Inflammation levels following strenuous exercise can be determined by measuring the level of C-reactive protein [16]. The effects of probiotics on endurance can be measured through the athlete’s oxygen uptake [17]. Even though many studies are focusing on the effects of probiotics on psychological conditions in healthy volunteers, limited approaches have been taken to study those effects on competitive athletes. Hence, this study is designed to determine the effects of daily probiotic supplementation on anxiety, stress, mood and fitness levels among competitive badminton players.

## 2. Materials and Methods

### 2.1. Research Design

This intervention was a randomized, placebo-controlled study. Recruited participants were randomly divided into the probiotic group (PG) and the control group (CG). The participants were given treatments for six weeks without altering the training schedule of the athletes. During the intervention, PG players were given daily a commercial probiotic drink that contained *Lactobacillus casei* at a dose of 3 × 10^10^ colony forming units (CFU) (80 mL/bottle) as claimed by the manufacturer and mixed with commercially available orange juice (120 mL). At the same time the CG players received commercially available orange juice (200 mL) as a placebo drink. Both probiotics and orange juice drinks are approved by the health authority (Ministry of Health, Malaysia).

Both groups received the same amount of the intervention drink (200 mL); where the drink’s color and smell are similar. In addition, the supplements were distributed to the players by an independent person that not involved in the study, which supports the double-blinded process.

Ethics approval to carry out this study was granted by the Research Ethics Committee of Universiti Teknologi MARA (UiTM) in Shah Alam, Selangor, Malaysia (600-IRMI 5/1/6 REC431/19). This study was conducted according to the guidelines of the International Declaration of Helsinki. Participation was voluntary and written consent was obtained from the players. Familiarization of each protocol was applied before the actual measurements were taken. The validity and reliability of the measurement were determined before data collection. Data was collected at two time-points, i.e., before the start of the nutritional intervention (baseline) and after six weeks. The players were not allowed to take any additional probiotic supplements during the study. However, they were allowed to continue their usual diet and supplementation. Their food intake was recorded and monitored using three-day records to detect any unusual dietary habits.

### 2.2. Participants

Thirty UiTM badminton players aged 18 to 30 were recruited in this study after getting their consent and fulfilling the inclusion criteria such as players were physically and mentally healthy and did not suffer from psychological disorders, chronic diseases (e.g., gastrointestinal disease, cardiovascular disease), acute injuries and intolerance to probiotics. All subjects were also non-smokers. All the players undergo the same training protocols (5 days per week for 2 h) with an exercise regimen (running, drills, skills, agility, speed, strength, tactical and interval training). The coach was also informed not to alter their training regime for the 6-week study duration substantially. With the regular ingestion of probiotics or placebo drink, respectively, it is envisaged that any apparent differences between the experimental and placebo groups would be primarily due to the supplement.

### 2.3. Anthropometric and Body Composition Measurement

Height was measured using a portable stadiometer (SECA model 213, Hamburg, Germany) to the nearest 0.1 cm. The body fat percentage, fat mass, and lean body mass were determined using the InBody 500 bioelectrical impedance analyser (InBody Co Ltd., Cerritos, CA, USA) at the baseline and after the intervention. Players were instructed to come to the laboratory after a three-hour fast and no prior exercise that day. All testing was performed in the morning and followed as per instruction given by the manual of Inbody 500. Players stood on the platform of the device barefoot with the soles of their feet on the electrodes. Then grasped the handles of the unit with their thumb and fingers to maintain direct contact with the electrodes. They stood still for ~1 min while maintaining their elbows extended fully and their shoulder joint abducted to approximately a 30-degree angle.

The validity and reliability of using a bioelectrical impedance analyser (BIA) have shown reliability with repeat measurements differing by less than 0.2% with a very small 95% CI [18]. The BIA showed excellent relative agreement to the estimated true value (ρ = 0.97 (0.96, 0.98)) when compared to BodPod (R^2^ = 0.88) and DXA (R^2^ = 0.92), but has wide limits of agreement (−4.25 to 8.37%) [18].

### 2.4. Anxiety, Stress and Mood

Anxiety level was determined using the revised competitive state anxiety inventory-2 (CSAI-2R) [19]. The inventory assessed three dimensions of anxiety, namely cognitive anxiety (five items), somatic anxiety (seven items), and self-confidence (five items), which was described using a four-point Likert scale, where one (1) is equal to “not at all” and four (4) is equal to “very much so”. The Cronbach alpha reliability coefficients for CSAI-2R were approximately 0.81 for cognitive anxiety, 0.81 for somatic anxiety, and 0.86 for self-confidence [19]. The validated Malay version of CSAI-2R was used in this study [20]. Distress was determined using the Leiden index of the depression sensitivity scale (LEIDS-r) questionnaire that consisted of 34 items of self-reported questions with reliability coefficients at 0.89 [21,22].

The perceived stress scale (PSS) questionnaire was used to identify the perception of stress among badminton players. The questionnaire contained ten (10) items that were rated using a five-point Likert scale, where 0 is equal to “never” and 4 is equal to “very often” with a reliability coefficient of 0.78 [23]. The validated Malay version of the PSS questionnaire was used for the study [24]. The Brunel mood scale (BRUMS) questionnaire, which was based on profiling mood state (POMS), was used in this study to determine the players’ moods [25,26]. The questionnaire comprised 24 items that assessed anger, confusion, depression, fatigue, tension and vigor with reliability coefficients of each subscale at 0.72, 0.70, 0.74, 0.70, 0.77 and 0.71, respectively. The score was determined using a five-point Likert scale, where 0 is equal to “not at all” and 4 is equal to “extremely” [26,27]. The Malay version, which was translated and validated, was used in the study [27].

### 2.5. Aerobic Capacity Measurement

The 20-m multi-stage shuttle run test was used to measure cardio-respiratory or aerobic capacity fitness. In brief, players were asked to run back and forth on a 20-m track at an initial speed of 8.5 km/h, which was gradually increased by 0.5 km/h every minute, by a pace dictated by a sound signal of a compact disc (20-m Shuttle Run test CD, Australian Sports Commission). The numbers of laps fully completed were recorded for each subject. Cardio-respiratory fitness was determined using the table of normative values by Ramsbottom et al. (1988) [28] and the predictive formula by Chia et al. (2005) [29].

### 2.6. Hand Strength and Leg Power Measurement

The handgrip test was used to determine hand strength. Dominant and non-dominant hand strength was determined using a digital hand dynamometer (Takei Scientific Instruments, Niigata, Japan). To measure the hand strength, the players stand upright position, and the measurement of handgrip strength was taken from a measuring arm positioned straight at a slight distance from and with no contact to the body [30]. Two attempts of the handgrip were performed with each hand, lasting 3 s each and separated by 60 s of recovery, from which the average value was calculated.

As for the leg power assessment (lower body power), the vertical jump was determined by measuring the distance of the most extreme point the subject could reach with their arm by jumping (soles entirely on the floor). In the standing position, jumping was performed by subjects to reach the highest possible point of touching distance [31]. The protocol was repeated, and the average jump distance was recorded with a recovery of 15 s for each attempt.

### 2.7. Speed and Agility Measurement

The 40-m dash was used to measure the players’ speed and, the t-test was used to measure agility. Speed is the maximum rate at which a person can move the body over a specific distance. In human performance terms, it refers to the speed of coordinated joint actions and whole-body movements. The test was conducted in open-space flat flor where a 40-m run is marked and a ’timing’ start line 10 m into the run. The subjects need to use a standing start to run the 40-m as quickly as possible—the time in seconds taken from the 10 m line to the 40 m line [32].

This t-test was used to measure agility. This test, in accordance with the nature of badminton, includes a forward sprint (9.14 m), then to the left side shuffle (4.57 m), then side-shuffle to the right (4.57 m), side-shuffle back to the left and back-peddled 9.14 m back to the start [32]. The players’ time was clocked with a stopwatch once the run was completed at the nearest value of 0.1 s. Players who crossed one foot in front of the other while shuffling or failing to reach the base, or failing to look upward during the test were considered to have failed in their attempt [32].

### 2.8. Food Record

The three-day dietary record (twice a week and once on weekends) was used to analyze the diet intake of the players, which profile the food intake during the intervention period. The portion sizes of the foods consumed were estimated using household measurements. A qualified nutritionist performed the diet analyze through the Nutritionist Pro^TM^ (Axxya Systems, Woodinville, WA, USA) software version 2.4.1 (First Data Bank INC., 2011) based on the Malaysian food database as reported previously [33].

### 2.9. Statistical Analysis

The study was based on comparing the means of two groups. Therefore, the paired *t*-test was used to analyse differences between pre-and post-intervention (Week 0 and Week 6), whilst the independent *t*-test was employed to analyse the differences between groups (PG and CG) (*p* < 0.05). Data were analysed using the IBM SPSS version 27.0 (IBM Corp, Armonk, NY, USA). A normality test was conducted to determine data distribution and confirm the use of parametric and non-parametric tests. Descriptive statistics were used to interpret demographic data and expressed in mean with standard deviation.

## 3. Results

### 3.1. Participants

Table 1 shows the physical characteristics and body composition of PG and CG players before the intervention. There were no significant differences in age, weight, height and body mass index (BMI) between groups at pre-intervention. PG and CG players also showed similar body fat percentage, fat and skeletal mass.

### 3.2. Body Composition Change

After six weeks of intervention, only minor changes were observed in BMI, body fat percentage, and fat mass in players from both groups (PG and CG) (Table 2). The PG players recorded a reduction in body fat, whereas CG showed some increment in fat mass. When compared between groups, the BMI and body composition of PG and CG players showed similarity at both pre-and post-intervention (Table 3).

### 3.3. Anxiety, Stress and Mood

Anxiety, stress, and mood were almost similar between PG and CG players at the beginning of the intervention. After six weeks, PG players recorded a significant anxiety reduction (*p* < 0.001) and stress (*p* < 0.001), but not for mood (Table 4). On the contrary, CG players did not show significant changes in any psychological variables. The anxiety and stress level of PG players after the intervention were also significantly lower than those of CG (Table 5).

### 3.4. Fitness Variables

PG and CG players had similar aerobic capacity, speed, agility, hand strength and leg power at the beginning of the study. However, after probiotic treatment, the aerobic capacity of PG players had significantly increased (*p* < 0.001) (Table 6). On the contrary, there were no changes in other fitness variables in both groups after the intervention. Results showed that all fitness variables between groups at the baseline and after treatment were similar (Table 7).

### 3.5. Energy and Macronutrient Intakes

Energy intake and macronutrients showed no significant difference between the two research groups at the baseline and the end of the study.

## 4. Discussion

The influence of probiotics on microbiota functions with overall health had been well studied. Gut microbiota had a symbiotic relationship with the host in many metabolic processes, such as fermentation of undigested carbohydrates, vitamin synthesis and lipid metabolism. Such processes could promote wellness in the host by enhancing certain functions, such as the immune system and cognitive ability. However, more evidence was still needed to elucidate the reciprocal interactions between probiotics and gut microbiota on benefiting athletes in terms of physical performance and exercise capacity.

This study showed that stress and anxiety in badminton players could be alleviated with the daily consumption of probiotics for six weeks. The findings in this study were strongly supported by Adikari et al. (2019), which observed a significant decrease in competitive anxiety and perceived stress among 20 football players after eight weeks of daily probiotic supplementation [33]. Moreover, the findings were also consistent with those reported by Allen et al. (2016), where stress and anxiety levels of a healthy population were observed to decrease after four weeks of probiotic supplementation [34].

In Japan, similar findings were also reported by Sashihara et al. (2013) and Sawada et al. (2017), even though these studies used different probiotic strains for different purposes. Sashihara et al. (2013) found that the anxiety experienced by university athletes could be reduced by taking probiotics daily for four weeks [35]. In Sawada et al. (2017), the study was not related to sport but on the effects of taking probiotics while undertaking complex academic subjects. The authors studied medical students undergoing an anatomy course that required them to perform autopsies, which was quite stressful for young first-timers. The authors observed that taking daily probiotics supplementation for four weeks provided the students with relief from mental stress and improved their quality of sleep [36].

Changes in stress and anxiety following the supplementation of probiotics might be explained by the underlying relationship between the microbiome and gut-brain axis (GBA) when gut microbiota respond to physical stress [11,37]. The GBA consisted of bidirectional communication between the central and enteric nervous system, bridging cognitive and emotional centers of the brain with peripheral intestinal functions. The interaction between microbiota and GBA worked through neural, endocrine, immune and humoral networks [38]. The bidirectional interaction formed the foundation of the causal effects of probiotic supplementation in reducing anxiety and stress when the intestinal microbiota interacted with the central nervous system, hence, affecting psychologically related symptoms and behavior in humans [39]. Microbiota could decrease stress-induced corticosterone secretion, reducing anxiety and depression-related behaviors by improving the brain expression of gamma-aminobutyric acid (GABA) receptors [40]. In contrast, pathogenic bacteria had been reported to induce anxiety-like behaviors by mediating vagal afferents [41]. Probiotics may also be capable of changing gut functions, thus triggering GBA responses by transforming dopamine into noradrenaline in the central and peripheral nervous systems, which would lead to a decrease in depressive behavior [42].

Another possible mechanism of microbiota for reducing anxiety is through the hypothalamic-pituitary-adrenal (H.P.A.) axis by enhancing hippocampal neurotrophic factor messenger expression R.N.A., which could decrease anxiety-like activity [43], which was demonstrated in a study that revealed improvements in subjects with stress-related disorders associated with HPA axis dysfunction, such as having a large proportion of glucocorticoid receptors and lower learning ability [44]. Probiotics were reported to transform dopamine into noradrenaline in the chromaffin cells of the adrenal medulla, resulting in a decrease in depression-related behavior [42].

The present study showed that the supplementation of probiotics did not affect the mood of badminton players, which was consistent with the evidence of a previous study by Kelly et al. (2017), which reported no influence and change in the mood and serum cortisol level of subjects, respectively, who were exposed to an acute stressor after eight weeks of *Lactobacillus rhamnosus* supplementation [45]. Benton et al. (2007) also proved that mood was not influenced by the consumption of *Lactobacillus casei* Shirota probiotic for three weeks [9].

The influence of probiotics on aerobic capacity in the present study was consistent with Mach and Fuster-Botella (2017), which reported an increase in oxygen uptake among swimmers who took probiotics [17]. In a previous study by Salarkia et al. (2013), a group of swimmers who consumed probiotics recorded a significant increase in maximal oxygen consumption (VO^2^max) compared to controls [46]. This same observation was observed by Salehzadeh (2015), who also reported the same outcome in the VO^2^max of his subjects [16].

There might be several explanations on how probiotic supplementation improved the aerobic capacity of athletes. Microbiota in the gut might play a role in the body’s energy system after a few minutes of muscle contraction, when the phosphocreatine concentration decreases, resulting in the need for other fuels. Gene expression for glycogenolysis would be induced to ensure ATP production for increased muscle activity requirements such as the cross-bridge cycle, myosin ATPase activity, and muscle ion pumps [47]. At the same time, consumption of free fatty acids for oxidation would also increase, enhancing not only the lipolysis of adipose tissues to meet the energy requirement, but also consuming other sources, such as fatty acids from microbiota activity. Thus, this would create a complicated and reciprocal relationship between the gut microbiota and the energy metabolism of the entire body [48], which was probably one of the mechanisms by which gut microbiota exerted beneficial effects on athletes’ performance.

Carbohydrate digestion is a core activity of the human gut microbiota, which drives energy and carbon metabolism in the colon, although the range of protein-generated end products is broader than carbohydrates [49]. In the colon, complex plant-derived polysaccharides, such as cellulose, *β*-glucan, xylan, mannan and pectin would be digested and subsequently fermented into short-chain fatty acids (SCFAs) by gut microorganisms, which were then used as carbon and energy sources by other specific bacteria such as reductive acetogens, sulfate-reducing bacteria and methanogens [50]. Modulation of microbiota might also enhance endurance in athletes and improvement of VO2max might be due to the prevention of upper respiratory tract infection [46]. Reduced anxiety and stress conditions due to probiotic supplements could improve athletes’ brain function, resulting in high performance in competitions [51].

Intense exercises had been associated with decreased intestinal permeability and its subsequent oxidative stress and inflammation. The gut microbiota, acting as a positive effector on gut health, could counter this effect by increasing intestinal permeability in trained athletes and reducing oxidative stress and inflammation [52]. Reactive oxygen species (ROS) is an essential indicator of oxidative stress, which was a contributing factor in the pathogenesis of gastrointestinal mucosal disorders [53]. Probiotic supplementation had demonstrated the alleviation of oxidative stress by reducing the formation of ROS [54]. In another related reaction, the production of catecholamines during exercise was another indicator of physical stress [55]. Probiotics-enriched gut microbiota could promote increased catecholamine activity and thus, minimize exercise-induced fatigue [56].

The present study had limitation that must be considered. The dietary intake control of certain food, i.e., indigestible polysaccharide, which may influence microbiome activity. Nevertheless, there were no changes in players’ habitual diet during the intervention. Objective measures of psycho-physiological may need to be considered in future studies.

## 5. Conclusions

Regular consumption of probiotics may confer benefits for sports athletes. This present study aimed to determine the effects of probiotic supplementation on anxiety, perceived stress, mood, and fitness of badminton players. Consuming probiotics might significantly help to alleviate competitive anxiety and stress, besides increasing the aerobic capacity of athletes. These findings suggest that probiotic supplements could be beneficial for athletes to enhance their mental state and physical performance. Probiotic supplementation that may influence the regulation of pathways (neuro-endocrine) and mechanism of action in response to physical and psychological stressors encountered by badminton players should further studied. Research evaluating psycho-physiological biomarkers in response to probiotic supplementation may provide greater insight for sports individuals.

## Figures and Tables

**Table 1:** Physical characteristic and body composition of PG and CG at the baseline.

	PG (*n* = 15)	CG (*n* = 15)	*p*-Value
Age (year)	19.5 ± 1.0	19.9 ± 1.3	0.274
Weight (kg)	63.9 ± 8.3	67.7 ± 7.1	0.191
Height (m)	1.66 ± 0.45	1.69 ± 0.61	0.128
BMI (kg/m^2^)	23.3 ± 3.2	23.6 ± 2.2	0.736
Body fat (%)	20.33 ± 6.35	18.36 ± 4.85	0.349
Fat mass (kg)	13.2 ± 5.7	12.4 ± 3.4	0.664

Note. Mean ± SD.

**Table 2:** Body mass index, body fat percentage and fat mass of PG and CG before and after 6-weeks of intervention.

Groups	Variables	Pre-Intervention	Post-Intervention	% Changes	* *p*-Value
PG (*n* = 15)	BMI (kg/m^2^)	23.3 ± 3.2	23.4 ± 2.6	0.4	0.732
	Body fat (%)	20.33 ± 6.35	20.23 ± 5.94	−0.5	0.720
	Fat mass (kg)	13.2 ± 5.7	13.1 ± 4.4	−0.4	0.877
CG (*n* = 15)	BMI (kg/m^2^)	23.6 ± 2.2	23.6 ± 2.2	0.0	0.821
	Body fat (%)	18.37 ± 4.85	18.53 ± 4.79	0.9	0.086
	Fat mass (kg)	12.4 ± 3.4	12.6 ± 3.3	1.6	0.183

Note. Mean ± SD, significant at *p* < 0.05 *.

**Table 3:** Comparison of body composition between PG and CG at pre- and post-intervention.

Timing	Variables	PG (*n* = 15)	CG (*n* = 15)	* *p*-Value
Pre-intervention (Week 0)	BMI (kg/m^2^)	23.3 ± 3.2	23.6 ± 2.2	0.736
	Body Fat (%)	20.33 ± 6.35	18.37 ± 4.85	0.349
	Fat Mass	13.2 ± 5.7	12.4 ± 3.4	0.664
Post-intervention (Week 6)	BMI (kg/m^2^)	23.4 ± 2.6	23.6 ± 2.2	0.804
	Body Fat (%)	20.23 ± 5.94	18.53 ± 4.79	0.395
	Fat Mass	13.1 ± 4.4	12.6 ± 3.3	0.728

Note. Mean ± SD, significant at *p* < 0.05 *.

**Table 4:** Anxiety, stress and mood of PG and CG before and after 6-weeks of intervention.

Groups	Variables	Pre-Intervention	Post-Intervention	% Changes	* *p*-Value
PG (*n* = 15)	Anxiety	26.47 ± 2.33	22.27 ± 2.40	15.9	<0.001 *
	Stress	23.27 ± 2.15	18.60 ± 1.40	20.1	<0.001 *
	Mood	51.27 ± 1.44	50.60 ± 1.76	1.3	0.259
CG (*n* = 15)	Anxiety	27.87 ± 1.64	26.33 ± 2.50	5.5	0.079
	Stress	23.53 ± 2.45	23.47 ± 2.20	0.2	0.836
	Mood	52.40 ± 1.76	51.80 ± 1.70	1.1	0.132

Note. Mean ± SD, significant at *p* < 0.05 *.

**Table 5:** Comparison of anxiety, stress and mood between PG and CG at pre- and post-intervention.

Timing	Variables	PG (*n* = 15)	CG (*n* = 15)	* *p*-Value
Pre-intervention (Week 0)	Anxiety	26.47 ± 2.33	27.87 ± 1.64	0.067
	Stress	23.27 ± 2.15	23.53 ± 2.45	0.754
	Mood	51.27 ± 1.44	52.40 ± 1.76	0.064
Post-intervention (Week 6)	Anxiety	22.27 ± 2.40	26.33 ± 2.50	<0.001 *
	Stress	18.60 ± 1.40	23.47 ± 2.20	<0.001 *
	Mood	50.60 ± 1.76	51.80 ± 1.70	0.068

Note. Mean ± SD, significant at *p* < 0.05 *.

**Table 6:** Aerobic capacity, speed, agility, hand strength and leg power of PG and CG before and after 6-weeks of intervention.

Group	Variables	Pre- Intervention	Post- Intervention	% Changes	* *p*-Value
PG (*n* = 15)	Aerobic Capacity (mL.kg^−1^.min^−1^)	66.01 ± 3.80	69.89 ± 4.33	5.88	<0.001 *
	Speed (s)	6.05 ± 0.07	6.06 ± 0.06	0.05	0.675
	Agility (s)	8.06 ± 0.06	8.04 ± 0.05	−0.15	0.138
	Hand Strength (kg)	56.20 ± 2.57	56.40 ± 2.13	0.36	0.705
	Leg Power (cm)	59.80 ± 1.82	60.40 ± 1.68	1.00	0.095
CG (*n* = 15)	Aerobic Capacity (mL.kg^−1^.min^−1^)	67.31 ± 4.31	67.93 ± 3.69	0.92	0.090
	Speed (s)	6.07 ± 0.07	6.07 ± 0.07	−0.02	0.900
	Agility (s)	8.03 ± 0.06	8.02 ± 0.06	−0.09	0.164
	Hand Strength (kg)	56.33 ± 1.72	56.53 ± 1.64	0.36	0.334
	Leg Power (cm)	58.93 ± 2.43	59.27 ± 1.98	0.56	0.290

Note. Mean ± SD, significant at *p* < 0.05 *.

**Table 7:** Comparison of aerobic capacity (VO_2_max), speed, agility, hand strength and leg power between PG and CG at pre- and post-intervention.

Timing	Variables	PG (*n* = 15)	CG (*n* = 15)	*p*-Value
Pre-intervention (Week 0)	VO_2_max (mL/kg/min)	66.01 ± 3.80	67.31 ± 4.31	0.388
	Speed (s)	6.05 ± 0.07	6.07 ± 0.07	0.632
	Agility (s)	8.06 ± 0.06	8.03 ± 0.06	0.322
	Hand Strength (kg)	56.20 ± 2.57	56.33 ± 1.72	0.868
	Leg Power (cm)	59.80 ± 1.82	58.93 ± 2.43	0.279
Post-intervention (Week 6)	VO_2_max (mL/kg/min)	69.89 ± 4.33	67.93 ± 3.69	0.192
	Speed (s)	6.06 ± 0.06	6.07 ± 0.07	0.700
	Agility (s)	8.04 ± 0.05	8.02 ± 0.06	0.391
	Hand Strength (kg)	56.40 ± 2.13	56.53 ± 1.64	0.849
	Leg Power (cm)	60.40 ± 1.68	59.27 ± 1.98	0.102

Note. Mean ± SD, significant at *p* < 0.05.

## Data Availability

The data is available upon request from the authors.
